# Examining the effects of *Salmonella* phage on the caecal microbiota and metabolome features in *Salmonella*-free broilers

**DOI:** 10.3389/fgene.2022.1060713

**Published:** 2022-11-10

**Authors:** Laura Lorenzo-Rebenaque, Cristina Casto-Rebollo, Gianfranco Diretto, Sarah Frusciante, Juan Carlos Rodríguez, María-Paz Ventero, Carmen Molina-Pardines, Santiago Vega, Clara Marin, Francisco Marco-Jiménez

**Affiliations:** ^1^ Department of Animal Production and Health, Veterinary Public Health and Food Science and Technology, Biomedical Research Institute, Faculty of Veterinary Medicine, Cardenal Herrera-CEU University, CEU Universities, Valencia, Spain; ^2^ Institute of Science and Animal Technology, Universitat Politècnica de València, Valencia, Spain; ^3^ Italian Agency for New Technologies, Energy and Sustainable Development (ENEA), Biotechnology Laboratory, Centro Ricerche Casaccia, Santa Maria di Galeria, Rome, Italy; ^4^ Microbiology Department, Balmis General University Hospital, Microbiology Division, Miguel Hernández University, ISABIAL, Alicante, Spain; ^5^ Microbiology Department, Balmis General University Hospital, ISABIAL, Alicante, Spain

**Keywords:** bacteriophages, poultry, omic sciences, high throughput sequencing, microbiome

## Abstract

Bacteriophages selectively infect and kill their target bacterial host, being a promising approach to controlling zoonotic bacteria in poultry production. To ensure confidence in its use, fundamental questions of safety and toxicity monitoring of phage therapy should be raised. Due to its high specificity, a minimal impact on the gut ecology is expected; however, more in-depth research into key parameters that influence the success of phage interventions has been needed to reach a consensus on the impact of bacteriophage therapy in the gut. In this context, this study aimed to investigate the interaction of phages with animals; more specifically, we compared the caecum microbiome and metabolome after a *Salmonella* phage challenge in *Salmonella*-free broilers, evaluating the role of the phage administration route. To this end, we employed 45 caecum content samples from a previous study where *Salmonella* phages were administered *via* drinking water or feed for 24 h from 4, 5 to 6-weeks-old broilers. High-throughput 16S rRNA gene sequencing showed a high level of similarity (beta diversity) but revealed a significant change in alpha diversity between broilers with *Salmonella*-phage administered in the drinking water and control. Our results showed that the phages affected only a few genera of the microbiota’s structure, regardless of the administration route. Among these, we found a significant increase in *Streptococcus* and *Sellimonas* in the drinking water and *Lactobacillus*, *Anaeroplasma* and *Clostridia_vadinBB60_group* in the feed. Nevertheless, the LC-HRMS-based metabolomics analyses revealed that despite few genera were significantly affected, a substantial number of metabolites, especially in the phage administered in the drinking water were significantly altered (64 and 14 in the drinking water and feed groups, respectively). Overall, our study shows that preventive therapy with bacteriophages minimally alters the caecal microbiota but significantly impacts their metabolites, regardless of the route of administration.

## 1 Introduction

Bacteriophage therapy is a promising approach to controlling zoonotic bacteria, replacing antibiotics to treat or prevent bacterial diseases in poultry production (Wernicki et al., 2017; Żbikowska et al., 2020; Clavijo et al., 2022; Zhao et al., 2022). Specifically, lytic bacteriophages (phages) are ‘natural predators’ that selectively infect and kill their target bacterial host (Mu et al., 2021; Zhao et al., 2022). Compared to antibiotics, phages have high specificity that usually attacks only their targeted bacterial hosts, indicating minimal disruption to the niche microbiota (Cieplak et al., 2018; Gindin et al., 2019). Nevertheless, phages targeting *Salmonella* can potentially lyse phylogenetically related genera, such as *Escherichia coli* or *Citrobacter* spp (Lorenzo-Rebenaque et al., 2021; Zhao et al., 2022). Different challenge experiments in poultry have revealed the efficacy of bacteriophage therapy application to control enteric pathogens (Carvalho et al., 2010; Nabil et al., 2018; Sevilla-Navarro et al., 2018; Clavijo et al., 2019; Richards et al., 2019; Huang et al., 2022). Till now, most phage-based products have been targeted against the main foodborne pathogens, such as *Campylobacter jejuni*, *Salmonella* spp, *Escherichia coli*, *Listeria monocytogenes*, *Staphylococcus aureus*, and *Clostridium perfringens* (Żbikowska et al., 2020). However, to ensure confidence in the use of phages, fundamental questions of safety and toxicity monitoring of phage therapy should be raised (Drilling et al., 2017; Krut and Bekeredjian-Ding, 2018; Dufour et al., 2019; Liu et al., 2021). Likewise, further research into crucial parameters that influence the success of phage interventions is needed to reach a consensus on the impact of bacteriophage therapy in the gut (Javaudin et al., 2021). In poultry, the data on the effects of phage treatment on dysbiotic events in gut microbiota are scarce (Clavijo et al., 2022; Zhao et al., 2022). Thus, before making any decisions on the use of phage as a therapy, our knowledge of phage-host interactions must be increased (Sutton and Hill, 2019). Bacteriophages have the potential to interact with the immune system directly (Ivanenkov and Menon, 2000; Barr et al., 2013, 2015; Nguyen et al., 2017), and given their larger size relative to other biological therapeutic agents, makes phages a more complex therapeutic agent than any biotherapeutics that have preceded them (Sutton and Hill, 2019). Even though ample research on bacteriophage therapy applications has provided many positive conclusions, there are still some unknowns regarding their role in gastrointestinal ecological homeostasis (Loc-Carrillo and Abedon, 2011).

The symbiotic interactions between host and gastrointestinal tract microbiota are fundamental to poultry health, as they have a positive impact on the immune system and broiler productivity (Brisbin et al., 2008). It is well known that gastrointestinal microbiota contributes to the reduction and prevention of enteric pathogen colonisation by competitive exclusion and the synthesis of bacteriostatic and bactericidal compounds in broilers (Clavijo and Flórez, 2018). Conversely, an unbalanced microbiota can induce several gut disorders, such as inflammation and leaky gut (Teague et al., 2017; Jacquier et al., 2019). Accordingly, a logical first step in exploring the safety of phage therapy would be to rule out that dysbiotic changes in the gut microbial community occur. Despite its ubiquity in the environment and its narrow range of action, the introduction of therapeutic phages to the microbiota will still act as an external agent (Hsu et al., 2019; Clavijo et al., 2022; Zhao et al., 2022). In this way, a recent report demonstrated that *Salmonella* phages induce changes in the intestinal microbiota of *Salmonella*-free chicks at early life stages (Zhao et al., 2022). In addition, in mammals it has been observed that exposure to a commercial bacteriophage preparation results in dysbiosis with increased inflammation and intestinal permeability (Tetz et al., 2017).

From this perspective, this study aimed to investigate the interaction of phages with the animal. More specifically, we compared the caecal microbiome and metabolome after a *Salmonella* phage challenge in *Salmonella*-free broilers, evaluating the role of the phage administration route.

## 2 Material and methods

### 2.1 Caecal content origin

The Directorate-General approved this study for Agriculture, Fisheries and Livestock from the Valencian Community (2021/VSC/PEA/0003). A total of 45 caecal content samples were obtained in a previous study on the use of phages in poultry, carried out at the Centre for Animal Research and Technology (CITA, IVIA, Segorbe, and Spain) (Lorenzo-Rebenaque et al., 2022). The animals involved in this study were *Salmonella*-free Ross male chicks (1-day-old), that purchased from a commercial hatchery and housed in the growing room under commercial rearing conditions on an experimental farm. Briefly, the house was supplied with wood shavings as bedding material, programmable electrical lighting, automated electric heating and forced ventilation. The environmental temperature was gradually reduced from 32°C on arrival day to 19°C at 39 days post-hatch (Montoro-Dasi et al., 2020). All animals had free access to food and water. Two different age commercial diets were offered to the animals, a pelleted starter diet from arrival until 21 days post-hatch (*Camperbroiler iniciación, Alimentación Animal Nanta, Spain*) and a pelleted grower diet from 21 days post-hatch to the slaughter day (*Pollos crecimiento G, Alimentación Animal Nanta, Spain*).

**FIGURE 1:**
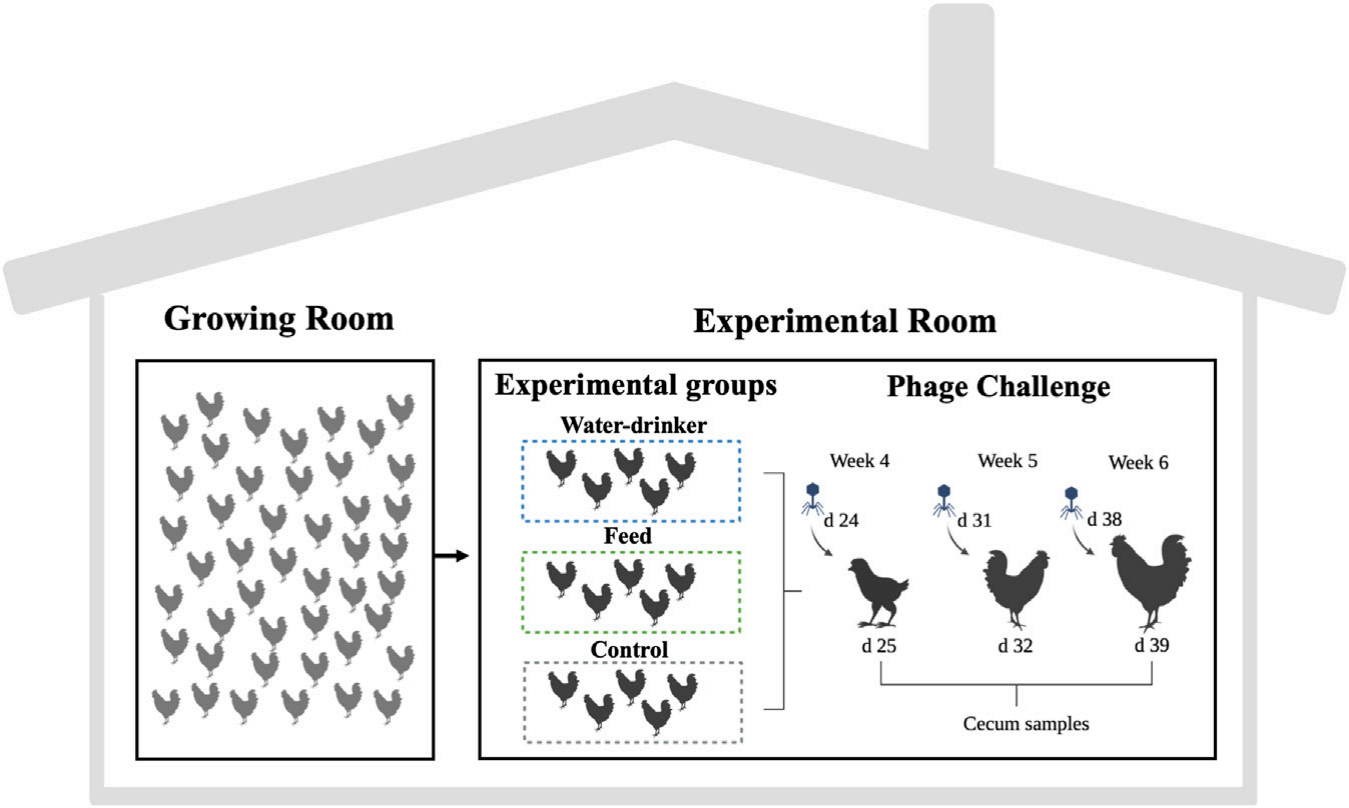
Experimental design of phage challenge. The water group received a 10^8^ PFU/ml phage concentration *via* drinking water. The feed group received a 10^8^ PFU/g phage concentration *via* feed (encapsulated). The control group did not receive a phage. Created with BioRender.com.

Weekly from week 4 to week 6 of the rearing period (fully competent immune system birds age), 15 birds were moved to another room (experimental room) and randomly divided into three groups, with 5 birds in each group (phages in drinking water -water group, phages in feed -feed group and no phages -control group) (Figure 1) (Lorenzo-Rebenaque et al., 2022). The phage used in this study is described in detail in Sevilla-Navarro et al. (2020) and Lorenzo-Rebenaque et al. (2021). The water group received a 10^8^ PFU/ml phage concentration *via* drinking water, the feed group received a 10^8^ PFU/g phage concentration *via* feed (encapsulated), and the control group did not receive a phage. Caecal samples were obtained 1 day after delivery of the phage. All animals of each group were slaughtered and the caecum was removed. Individual caecal content was divided into two flash-frozen aliquots in liquid nitrogen for subsequent microbiome and metabolome analyses.

### 2.2 Microbiota analysis

#### 2.2.1 DNA extraction, 16S rRNA gene amplification and MiSeq sequencing

First, caecal content was removed and homogenised. Then, the DNA was extracted from 250 mg of each sample according to the manufacturer’s instructions (QIAamp Power Fecal Pro DNA kit, Werfen, Barcelona, Spain). DNA concentration and purity were measured using a Nanodrop ND-1000 spectrophotometer (Thermo Scientific, Wilmington, United States), and verified using a Qubit fluorometer (Life Technologies, Paisley, UK). The DNA was frozen at −20°C for shipment at the *Instituto de Investigación Sanitaria y Biomédica de Alicante—ISABIAL* (Alicante, Spain), following the manufacturer’s instructions. Once there, 16S rRNA gene amplification and MiSeq sequencing was performed. To this end, from 12.5 ng of DNA (evaluated in Qubit) of each sample, the library was prepared following the instructions of the 16S rRNA Metagenomic Sequencing Library Preparation (Illumina) protocol (Illumina, 2022). Primer sequences cover the V3 and V4 regions of the 16S rRNA gene. The following primers also include the Illumina adapters: 16S Amplicon PCR Forward Primer = 5′ TCGTCGGCAGCGTCAGATGTGTATAAGAGACAGCCTACGGGNGGCWGCAG; and 16S Amplicon PCR Reverse Primer = 5′ GTCTCGTGGGCTCGGAGATGTGTATAAG AGACAGGA CTACHVGGGTATCTAATCC. NGS Libraries were analysed using the Agilent 4200 TapeStation System to ensure their integrity. The sequencing run was performed in a MiSeq (Illumina) system in 2 × 300 bp format. The quality of the raw unprocessed reads was evaluated using the FastQC software (Bioinformatics, 2022).

#### 2.2.2 Bioinformatic analysis

To perform the bioinformatic analysis, demultiplexed paired FASTQ sequences were imported into the QIIME2 v2021.4 (Bolyen et al., 2019). The DADA2 pipeline incorporated into QIIME2 was used for the denoising, filtering and chimera removal of the sequences and assigned reads into Amplicon Sequence Variants (ASVs). Then, taxonomic annotation was obtained using the SILVA v138 database (Quast et al., 2013; Campos et al., 2022), and sequences not assigned to any taxa or classified as eukaryote, archaea or only bacteria were filtered out. Sequencing statistical analyses were done using QIIME2 v2021.4. The datasets generated and analysed are available at NCBI’s BioProject PRJNA876127.

### 2.3 Metabolomics analysis

#### 2.3.1 Sample preparations

The sample preparations were performed according to previous published method with slight modifications (Coppola et al., 2019). Briefly, caecal contents were lyophilised and homogenised. Then, 10 mg of the sample was mixed with 0.75 ml of cold 75% (v/v) methanol and 0.1% (v/v) formic acid, spiked with 10 μg/ml formononetin as internal standard, the mix was shaken for 40’ at 20 Hz using a Mixer Mill 300 (Qiagen) and centrifuged at 20,000 xg for 15 min at 4°C. The suspension (600 μL) was transferred to a new 2-ml conical tube. For the LC-ESI-MS analysis, samples were transferred to HPLC tubes and an aliquot of 3 μl was injected. Finally, the supernatant was collected, filtered with HPLC filter tubes (0.22 µm pore size, WhatmannTM) and 3 μl were subjected to LC-ESI-HRMS analysis using an LTQ-Orbitrap Discovery mass spectrometry system (Thermo Fisher Scientific) as previously described (Garcia-Dominguez et al., 2020).

#### 2.3.2 LC-ESI-HRMS analysis

Untargeted LC-ESI-HRMS analyses of the caecal semipolar metabolome were performed as reported above (Garcia-Dominguez et al., 2020) in the *Agenzia nazionale per le nuove tecnologie, l’energia e lo sviluppo economico sostenibile* (ENEA, Roma, Italy). Compound Discoverer software (Thermofisher Scientific) was used to identify the differentially accumulated peaks, by chromatogram alignment and peak alignment/picking/filtration, and public database (e.g., ChemSpider, KEGG, Metabolika) querying based on accurate masses (m/z). After chromatogram alignment and retrieval of all the detected frames (e.g., ions), the data generated were normalised with respect to the internal standard. For metabolite identification, a manual curation using the Metlin database was performed (https://metlin.scripps.edu/). Tentative identifications were validated comparing chromatographic and spectral properties with authentic standards (when available) and reference spectra, in house database, literature data, and based on the m/z accurate masses, as reported in the Pubchem database (http://pubchem.ncbi.nlm.nih.gov/) for monoisotopic mass identification, subsequently confirmed by MS/MS fragmentation. This data is available at the NIH Common Fund’s National Metabolomics Data Repository (NMDR) website, the Metabolomics Workbench, https://www.metabolomicsworkbench.org where it has been assigned study ID ST002310.

### 2.4 Statistical analysis

Statistical analysis of microbiome and metabolome composition was performed following the same methodology. No outlier samples were identified using a principal component analysis with the dataset without zeros, so all samples remained in the datasets. Genera and metabolites with more than 20% zeros within each treatment were removed (Bijlsma et al., 2006). The remaining zeros were replaced by one for microbiome data and by half of the minimum value detected for each metabolite. A total of 124 genera from 44 samples and 718 metabolites from 37 samples remained in the datasets. Datasets were transformed using the additive log-ratio (ALR) transformation following:

$$ALR\left(j|ref\right)=\log\left(\frac{x_{j}}{x_{ref}}\right)=\log\left(x_{j}\right)-\log\left(x_{ref}\right)$$ (1)

where **j** is the total number of variables in the dataset, $x_{j}$ is the values for the genera or metabolite j, and $\left(x_{ref}\right)$ is the reference variable used to transform the data. The reference variable for metabolome data was a standard chemical (formononetin) injected in the platform run at a fixed concentration. For microbiome data, $x_{ref}$ was the one with the lowest coefficient of variation ( $x_{ref}$ ; *Anaerofustis*). The lack of isometry was checked using a Procrustes correlation analysis (Greenacre et al., 2021). ALRs were auto scaled with mean of 0 and standard deviation of 1.

A partial least square-discriminant analysis (PLS-DA) was used to identify the genera and metabolites that allow classification or discrimination among the treatments. PLS-DA models were computed with the mixOmics packages in R (Rohart et al., 2017), using the treatments as the categorical vector y, and the ALR dataset for genera or metabolites as the matrix X. The balance error rate (BER) for the Mahalanobis distance, computed by a 4-fold cross-validation repeated 100 times was used to select the optimal number of components of the model in each iteration process. In each iteration, variables with a variable importance prediction (VIP) lower than one were removed from the X matrix, as they are not informative for the classification among the treatments (Galindo-Prieto et al., 2014). After the variable selection, a new PLS-DA model was computed. Variable selection and the PLS-DA model computation were done until the lowest BER was achieved, meaning that the best classification and prediction performance was achieved for the model. The prediction performance of the final PLS-DA model was checked with the construction of a confusion matrix and a permuted-confusion matrix using a 4-fold cross-validation repeated 10,000 times. The former allows us to determine the ability of the model to predict each treatment according to the variables selected by the PLS-DA. The latter determines if the performance achieved is due to a spurious selection of variables throughout the PLS-DA iterations. The prediction performance was considered spurious when the percentage of true positives for each treatment was far from their random probabilities (33% for three categories and 50% for two categories).

Bayesian statistics were used complementary to the PLS-DA to measure the relevance of the differences in abundance of genera and metabolites between the treatment (drinking water and feed groups) and the control group. Hence, a model with a single effect of ‘treatment’ and flat priors was fitted. The marginal posterior distribution of the unknows was done with MCMC (Gibbs sampling) using four chains with a length of 50,000 iterations, a lag of 10, and a burn-in of 1,000 interactions. The posterior mean of the differences in genera or metabolites abundances was estimated as the mean of the marginal posterior distribution of differences between the control and each of the treatments. These differences were estimates were reported as units of standard deviations (SD) of each genus or metabolite. The differences in the mean abundance of the genera and metabolites between the control and the treatments were considered relevant when these differences were higher than 0.5 units of SD, and the probability of the differences (Blasco, 2017) being higher (if the difference is positive) or lower (if negative) than 0 (P0) was higher than 0.9.

The alpha- and beta diversity were computed using the ALR at species level to measure the differences in microbiome composition among groups. The alpha diversity was measured by Shannon’s (H′) and inverse Simpson indexes to analyse the species diversity and evenness. Differences in the distribution of alpha diversity among groups were considered when the p-value of a Mann-Whitney U test was lower than 0.05. Beta diversity was measured by the Bray-Curtis dissimilarity matrix and a nonmetric multidimensional scaling (NMDS) was carried out to retrieve the loadings of the first two dimensions. Differences in microbial genera composition were tested by the permutational multivariate analysis of variance (PERMANOVA; p-value  <  0.05) on the loadings of the two first MDS dimensions.

## 3 Results

### 3.1 Changes in caecal microbiota

The caecal microbiota was characterised in 44 samples from the three groups (15 of the water group, 14 of the feed group and 15 of the control group) taken after 24 h of phage application at weeks 4, 5 and 6 of the chickens’ rearing period. The total of sequencing reads was 7,044,611 (average 156,546.9 reads/sample), with an average read length of 404.5 ± 14.79 pb. A total of 4,192,062 sequences and 2,778 ASVs were generated. A total of 4,144,140 sequences were left for ASVs table generation and database alignment (Supplementary Table S1). After filtering, a total of 2,735 ASVs were left for taxonomic assignment.

**FIGURE 2:**
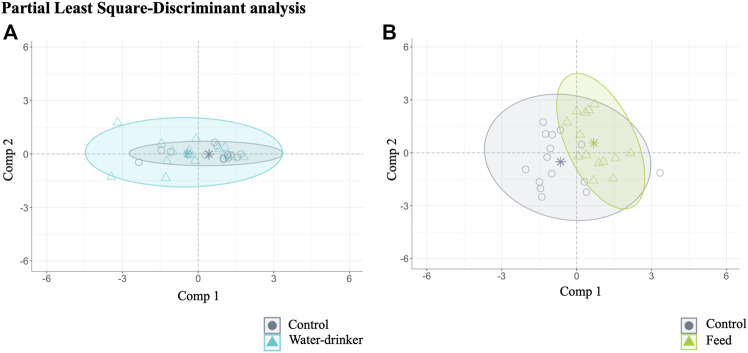
Caecal microbiota features characterised for *Salmonella*-free broilers treated with a *Salmonella* phage. Caecal microbiota composition dissimilarity through the representation of the first (Comp 1) and second components (Comp 2) of the identified 11 relevant variables (genera) in the final partial least square-discriminant analysis (PLS-DA) models **(A)** from the water *vs.* control groups, and **(B)** from the feed *vs.* control groups. The water group (blue) received a 10^8^ PFU/ml phage concentration *via* drinking water. The feed group (green) received a 10^8^ PFU/g phage concentration *via* feed (encapsulated). The control (grey) group did not receive a phage.

A PLS-DA with ALR transformed variables were used to evaluate the effect of the administration route (drinking water and feed groups) in the caecal microbial abundance in *Salmonella*-free broilers. The analysis identified 11 relevant variables (genera) in the final model: two for water *vs.* control group (final PLS-DA model classification performance: water = 59.83% and control = 71.99%, Figure 2A), and seven for feed *vs.* group (final PLS-DA model classification performance: feed = 74.13% and control = 77.91%, Figure 2B). Overall, the results show that a few genera (2 and 7) were the most potential to discriminate the effect of the phage administration.

**FIGURE 3:**
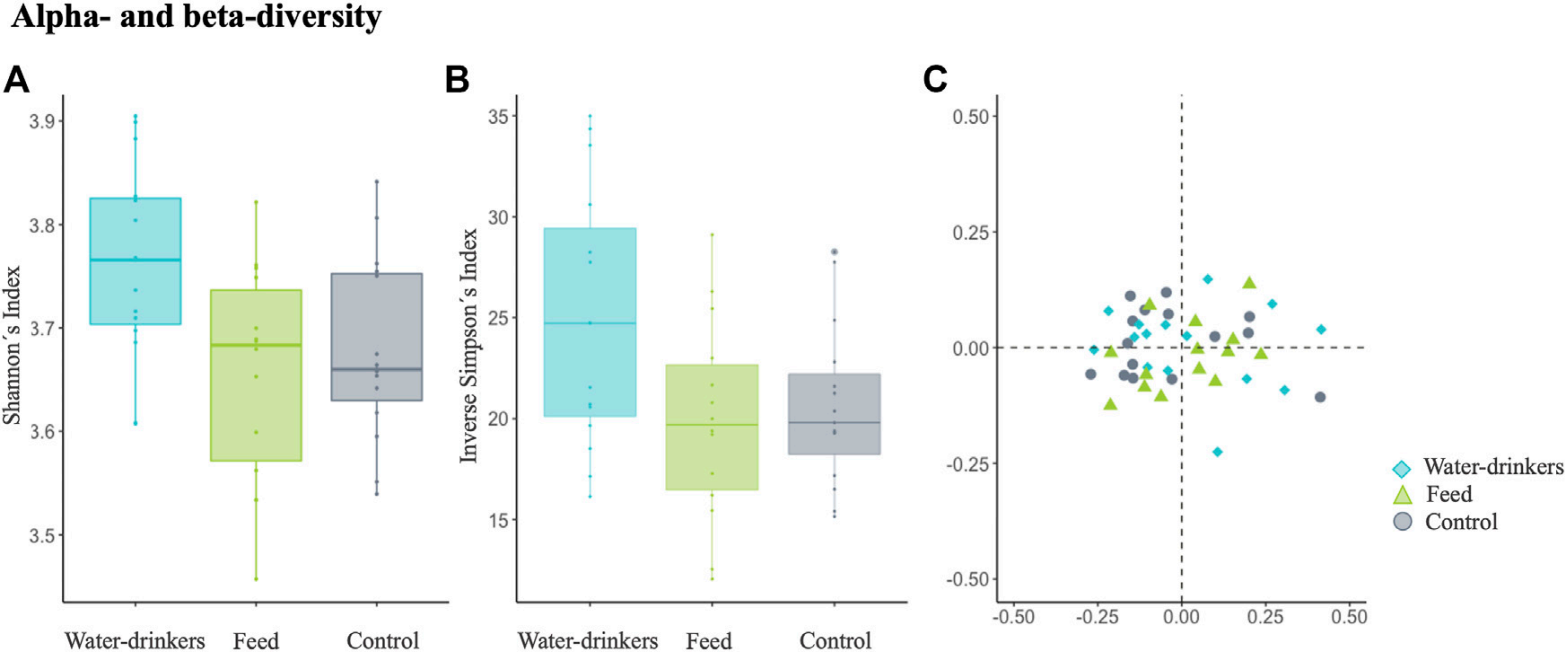
Examining the effects of *Salmonella* phage on the caecal microbiota in *Salmonella*-free broilers. Caecal microbiota composition dissimilarity through the representation of the and alpha- and beta diversity scores from water, feed and control groups. The alpha- and beta diversity scores were calculated with the additive log-ratio of each species abundance according to a reference genus (*Anaerofustis*). Alpha diversity was computed using **(A)** Shannon’s H index and **(B)** Inverse Simpson index. Beta diversity was computed by calculating **(C)** the Bray-Curtis dissimilarity matrix. Differences among populations were established with a p-value ≤ 0.05. The water group (blue) received a 10^8^ PFU/ml phage concentration *via* drinking water. The feed group (green) received a 10^8^ PFU/g phage concentration *via* feed (encapsulated). The control (grey) group did not receive a phage.

The Shannon diversity index, which is more sensitive to species richness, showed that the microbiota diversity of the water group was significantly different from that of the control and feed groups (Kruskal–Wallis test, water *vs.* control: p-value = 0.02, and feed *vs.* control: p-value = 0.78, Figure 3A). For the inverse Simpson index, which is more sensitive to species evenness, no significant differences were observed between groups (Kruskal-Wallis test, water *vs.* control: p-value = 0.07, feed *vs.* control: p-value = 0.81, Figure 3B). Moreover, in pairwise PERMANOVA comparisons between groups using Bray-Curtis, there were no significant differences between groups in the microbiome composition (p-value = 0.559; Figure 3C). These results showed that despite the few genera identified by PLS-DA, they are enough to show differences in alpha diversity in the water group. On the other hand, for the feed and control group, in general both populations displayed a similar microbiome composition, except for the seven relevant genera identified by the PLS-DA.

A Bayesian statistical analysis was performed to better understand the effect of *Salmonella* phage on the caecal microbiota from the initial relevant genera identified by PLS-DA. The Bayesian results showed that few of the variables included in the PLS-DA model were key variables for discriminating between groups, with relevant differences in mean abundance (Supplementary Table S2).

As seen in Table 1, in the water group, two of the 124 genera detected were different from those of the control group: *Streptococcus* and *Sellimonas*. Both genera, indeed, were more abundant in the water group, and were from the *Firmicutes* phyla. As seen in Table 1 **,** in the feed group, three of the 124 genera detected were different from those of the control group: *Lactobacillus, Anaeroplasma* and *Clostridia_vadinBB60_group*. All genera were more abundant in the feed group and were from the *Firmicutes* phyla.

**TABLE 1:** Caecal microbiota features characterised for *Salmonella*-free broilers treated with a *Salmonella* phage. Key genera identified by partial least square-discriminant analysis (PLS-DA) for discriminating between groups with relevant differences in mean abundance based on Bayesian statistical analysis in water- and feed-phage treated chickens compared with the control group, computed as control *vs.* water and control *vs.* feed. The water group received a 10^8^ PFU/ml phage concentration *via* drinking water. The feed group received a 10^8^ PFU/g phage concentration *via* feed (encapsulated). The control group did not receive a phage.

Experimental groups	Family	Genera	HPD95	P0	D
Control *vs*. Water	*Streptococcaceae*	*Streptococcus*	[-1.34.0.14]	94.97	-0.62
	*Lachnospiraceae*	*Sellimonas*	[-1.34.0.12]	94.73	-0.60
Control *vs*. Feed	*Lactobacillaceae*	*Lactobacillus*	[-1.32.0.18]	93.39	-0.57
	*Acholeplasmataceae*	*Anaeroplasma*	[-1.62,-0.17]	99.11	-0.89
	*Clostridia_vadinBB60_group*	*Clostridia_vadinBB60_group*	[-1.45.0.03]	96.87	-0.70

HPD95%, The highest posterior density region at 95% of probability; P0, Probability of the difference (Dcontrol-water or Dcontrol-feed) being greater than 0 when Dcontrol-water or Dcontrol-feed > 0 or lower than 0 when Dcontrol-water or Dcontrol-feed < 0; D, Mean of the difference control *vs.* water or control *vs.* feed (median of the marginal posterior distribution of the difference between the control group and the water group or feed group). Statistical differences were assumed if | Dcontrol-water | or | Dcontrol-feed | surpass R value and its P0 > 0.90.

### 3.1 Changes in caecal metabolome

The caecal metabolome included 37 samples, namely 12 individuals in the water group, 13 individuals in the feed group and 12 individuals in the control group, obtained after 24 h of phage application at weeks 4, 5, and 6 of the chickens’ growth phase. First of all, an untargeted LC–HRMS-based metabolomics pipeline was used to analyse the metabolic regulation in phage-treated chickens (water and feed-treated). In this way, a total of 717 peaks were retained.

**FIGURE 4:**
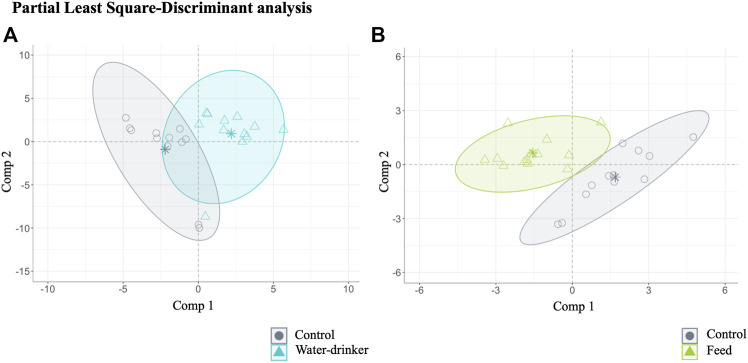
The caecal metabolome features characterised for *Salmonella*-free broilers treated with a *Salmonella* phage. Caecal metabolome composition dissimilarity through the representation of the first (Comp 1) and second components (Comp 2) of the identified 70 relevant variables (metabolites) in the final partial least square-discriminant analysis (PLS-DA) models **(A)** from the water *vs.* control groups, and **(B)** from the feed *vs.* control groups. The water group (blue) received a 10^8^ PFU/ml phage concentration *via* drinking water. The feed group (green) received a 10^8^ PFU/g phage concentration *via* feed (encapsulated). The control (grey) group did not receive a phage.

A PLS-DA with ALR transformed variables were used to evaluate the effect of the administration route (drinking water and feed groups) in the caecal metabolome variations of *Salmonella*-free adult broilers. Overall, the analysis identified 70 relevant variables (metabolites) in the final model: 64 for water compared with the control group (final PLS-DA model classification performance: water = 86.90% and control = 84.15%, Figure 4A), and 14 for feed compared with the control group (final PLS-DA model classification performance: feed = 97.24% and control = 93.92%, Figure 4B). Notably, only eight metabolites were common to both administration routes. The results showed, thus, that after phage administration, regardless of the administration route, some metabolites (64 and 14 from 717 metabolites identified for water and feed groups, respectively) were the most potential to discriminate the effect of the phage administration.

We further verified the relevant metabolites identified by PLS-DA and Bayesian statistical analysis, with showed that 27 variables from the initial 64 identified for water compared with the control group and 14 from the initial 14 identified for feed compared with the control group by PLS-DA analysis (Supplementary Table S3) had a posterior mean of the differences of at least 0.5 of the SD of the variable, in which the probability of differences being higher or lower than 0 (P0) was higher than 0.90.

For the water group, 16 of the 27 significant metabolites were down-regulated and 11 were up-regulated compared to the control group. Of these, 14 could be tentatively identified. The structures of the identified metabolites included organic acids and derivates (6), organic oxygen compounds (3), phenylpropanoids and polyketides (2), and Benzenoids (2), and organ heterocyclic compounds (1) (Table 2). For the feed group, 5 of the 14 significant metabolites were down-regulated and nine were up-regulated compared to the control group. Of these, 6 could be tentatively identified. The structures of the identified metabolites included lipids and lipid-like molecules (2), organic oxygen compounds (2), organic acids and derivates (1), and phenylpropanoids and polyketides (1) (Table 2).

**TABLE 2:** Caecal metabolome features characterised for *Salmonella*-free broilers treated with a *Salmonella* phage. Key metabolites identified by partial least square-discriminant analysis (PLS-DA) for discriminating between groups with relevant differences in mean abundance based on Bayesian statistical analysis in water- and feed-phage treated chickens compared with the control group, computed as control *vs.* water and control *vs.* feed. The water group received a 10^8^ PFU/ml phage concentration *via* drinking water. The feed group received a 10^8^ PFU/g phage concentration *via* feed (encapsulated). The control group did not receive a phage.

Experimental groups	Super class	Class	Subclass	Name	HPD95	P0	D
Control *vs.* Water	Organic oxygen compounds	Organooxygen compounds	Carbohydrates and carbohydrate conjugates	Dimboa glucoside	[-1.57.0.02]	96.95	-0.76
			Carbonyl compounds	Stearyl monoglyceridyl citrate	[-0.12.1.52]	95.24	0.70
				2-Hydroxy-4-methoxyacetophenone 5-sulphate	[-0.16.1.47]	93.64	0.63
	Organic acids and derivates	Peptidomimetics	Hybrid peptides	L-beta-aspartyl-L-alanine	[-0.06.1.59]	96.22	0.73
		Carboxylic acids and derivates	Amino acids, peptides and analogues	Tolmetin glucuronide	[-0.08.1.55]	96.13	0.73
				Hydroxyphenylacetylglycine	[-0.2.1.45]	93.54	0.63
				Nicotinamide Adenine Dinucleotide Phosphate	[-1.36.0.31]	90.70	-0.55
			Carboxylic acid derivates	N-Acetylcadaverine	[-0.04.1.55]	97.30	0.78
	Lipids and lipid-like molecules	Prenol lipids	Monoterpenoids	Monomenthyl succinate	[-0.2.1.47]	93.64	0.64
		Fatty Acyls	Eicosanoid	PGA3	[-1.55.0.1]	95.40	-0.70
	Benzenoids	Phenols	Phenol ethers	Dictagymnin	[-0.12.1.55]	95.71	0.72
		Benzene and substituted derivates	Benzene and substituted derivates	4-Methyl-1-phenyl-2-pentanone	[-0.13.1.53]	95.85	0.73
	Phenylpropanoids and polyketides	Cinnamic acids and derivatives	Hydroxycinnamic acids and derivatives	(R)-2-Feruloyl-1-(4-Hydroxyphenyl)-1,2-ethanediol	[-1.68,-0.15]	98.93	-0.92
		Kavalactones	Kavalactones	5,6-Dihydro-11-methoxyyangonin	[-1.42.0.26]	90.94	-0.56
	Non-identified metabolite 150				[−1.48.0.19]	93.99	−0.66
	Non-identified metabolite 152				[−1.44.0.22]	93.34	−0.63
	Non-identified metabolite 203				[−1.68,−0.09]	98.67	−0.89
	Non-identified metabolite 213				[−1.57.0.02]	97.29	−0.77
	Non-identified metabolite 233				[−1.57.0]	97.59	−0.79
	Non-identified metabolite 273				[−1.94,−0.57]	99.96	−1.25
	Non-identified metabolite 326				[−0.03.1.62]	97.07	0.79
	Non-identified metabolite 400				[0.21.1.78]	99.35	1.00
	Non-identified metabolite 566				[−0.3.1.37]	91.27	0.57
	Non-identified metabolite 568				[−0.08.1.56]	95.68	0.71
	Non-identified metabolite 572				[−0.09.1.56]	96.34	0.76
	Non-identified metabolite 573				[−0.3.1.35]	90.15	0.54
	Non-identified metabolite 678				[−0.04.1.6]	97.16	0.80
Control *vs.* Feed	Organic oxygen compounds	Organooxygen compounds	Carbohydrates and carbohydrate conjugates	Dimboa glucoside	[−1.47.0.1]	96.08	−0.70
			Carbonyl compounds	4-(2-Aminophenyl)-2,4-dioxobutanoic acid	[−1.44.0.16]	93.74	−0.63
	Organic acids and derivates	Carboxylic acids and derivates	Amino acids, peptides and analogues	L-Agaritine	[0.17.1.71]	99.23	0.96
	Lipids and lipid-like molecules	Steroids and steroid derivates	Sulphated steroids	Androsterone sulphate	[−1.54.0.04]	96.57	−0.74
		Prenol lipids	Quinone and hydroquinone lipid	7C-aglycone	[−1.48.0.12]	95.20	−0.68
	Phenylpropanoids and polyketides	Cinnamic acids and derivatives	Hydroxycinnamic acids and derivatives	(R)-2-Feruloyl-1-(4-Hydroxyphenyl)-1,2-ethanediol	[−1.65,−0.12]	98.86	−0.90
	Non-identified metabolite 154				[0.59.1.9]	99.97	1.24
	Non-identified metabolite 203				[−1.5.0.05]	96.43	−0.71
	Non-identified metabolite 213				[−1.61,−0.06]	98.63	−0.88
	Non-identified metabolite 233				[−1.64,−0.11]	98.85	−0.89
	Non-identified metabolite 273				[−2.04,−0.7]	99.98	−1.36
	Non-identified metabolite 400				[−0.12.1.41]	94.89	0.63
	Non-identified metabolite 420				[−0.04.1.54]	96.65	0.75
	Non-identified metabolite 557				[−0.16.1.46]	94.79	0.66

HPD95%, The highest posterior density region at 95% of probability; P0, Probability of the difference (Dcontrol-water or Dcontrol-feed) being greater than 0 when Dcontrol-water or Dcontrol-feed > 0 or lower than 0 when Dcontrol-water or Dcontrol-feed < 0; D, Mean of the control *vs.* water or control *vs.* feed difference (median of the marginal posterior distribution of the difference between the control group and the water group or feed group). Statistical differences were assumed if | Dcontrol-water | or | Dcontrol-feed | surpass R value and its P0>0.90.

## 4 Discussion

Bacteriophages are considered as a potent biocontrol agent to control zoonotic bacteria replacing antibiotics in poultry production (Wernicki et al., 2017; Zbikowska et al., 2020; D'Angelantonio et al., 2021; Clavijo et al., 2022; Zhao et al., 2022). In fact, the US food safety and inspection service allows the use of *Salmonella*-specific phage against bacterial contamination in live poultry before processing (Huff et al., 2002; Zhang et al., 2019). Nevertheless, although several challenge experiments in poultry have revealed the efficacy of phage therapy to control enteric pathogens (Carvalho et al., 2010; Nabil et al., 2018; Sevilla-Navarro et al., 2018; Clavijo et al., 2019; Richards et al., 2019; Huang et al., 2022), few studies have evaluated the role of bacteriophage exposure in animals and the possible effects on gut physiology (Tetz and Tetz, 2016; Tetz et al., 2017; Hsu et al., 2019; Zhao et al., 2022). Gut microbiota has been recognised as a hidden ‘metabolic organ’, with a great impact on host biological functions with long-term physiological effect (Robinson et al., 2022).

In this respective, the results of this study showed that the application of phages did not modulate the caecal microbiota beta diversity in target bacteria-free chickens. This fact is expected based on the nature of the bacteriophages, as a virus that has one-to-one correspondence with specific bacteria (Loc-Carrillo and Abedon, 2011). Notably, when phage was administered in the drinking water, microbial alpha diversity was altered. Overall, we detected an increase in the richness and diversity of caecal microbiota, indicating that the total number of bacterial species increased after phage treatment compared to the baseline pre-treatment data. Previous authors also reported an increase in the Shannon index after the *Salmonella*-phage application on animals free of the target bacteria (Tetz et al., 2017; Zhao et al., 2022). Zhao et al. (2022) showed that its application during the establishing and development of the intestinal microbiota in the first stages of the chicken production cycle leads to the greatest effect of the phage. Nevertheless, our results displayed that these changes could also take place in the later stages. The differences between the two groups may be because encapsulation and delivery methods delay the phage effect (Colom et al., 2017), and microbiota has been considered temporally stable with a dynamic equilibrium, with alterations that have complex and difficult to predict responses and consequences (Tetz et al., 2017).

Nevertheless, we found a minimal modification in the relative abundance of some microorganisms, regardless of the phage administration route. This result was consistent with previous studies (Tetz et al., 2017; Hsu et al., 2019; Zhao et al., 2022). Phage predation on non-targeted species has been reported previously. Different theories seek to shed light on these phenomena, such as the molecular changes, as single amino acid substitutions and unusual homologous intragenomic recombination that could promote the viral host jump and the diversification of the phage-host spectrum (de Sordi et al., 2017). Notably, the abundance of *Streptococcus* and *Sellimonas* was higher when phages were applied *via* drinking water. *Streptococcus* is a common microorganism found in the gastrointestinal tract of poultry (Yadav and Jha, 2019). However, higher abundance of this genus may not be of interest, as it could cause diseases in broilers and has been negatively correlated with body weight (Thibodeau et al., 2015; Lundberg et al., 2021). Conversely, a higher abundance of *Sellimonas* has been reported to be involved in recovered intestinal homeostasis after dysbiosis events (Muñoz et al., 2020). The abundance rates of *Lactobacillus*, *Anaeroplasma* and *Clostridia_vadinBB60_group* were higher when phages were included in the feed. The *Lactobacillus* genus is part of the group of commensal bacteria that function on vitamin production and antibacterial properties (Wang et al., 2014; Rodrigues et al., 2020). A higher relative abundance of *Anaeroplasma* was also reported in broilers after essential oil administration (Chen et al., 2020), and it has been reported to be positively corrected with the digestibility in other livestock production animals (Zhong et al., 2021). Moreover, an increased relative abundance of *Anaeroplasma* was also related to stressors in the chicken caeca, such as treatments against *Campylobacter* (Ty et al., 2022). Finally, we also found higher representativeness of *Clostridia_vadinBB60_group*, which is considered one of the most dominant microorganisms in the caecum, with an essential role in carbohydrate fermentation and short-chain fatty acid production (Memon et al., 2022).

However, we observed that these few altered genera significantly impact the caecum metabolome, with the most significant effect when phage was administered in the drinking water, and particularly affecting organic acids, lipid metabolism, and organic oxygen compounds notably. Overall, this result is consistent with previous studies on altered metabolites after gastrointestinal therapy, such as lipids and lipid-like molecules, organic acids and organoheterocyclic compounds (Li et al., 2018; Chen et al., 2020; Wu Y. et al., 2021a; Tang et al., 2021).‬ Indeed, phage predation could knock down associated metabolic products (Hsu et al., 2019). In this sense, Han et al. (2022) showed the metabolic changes occurring in *Klebsiella pneumoniae* following phage application, by reporting alterations in the metabolism of amino acids and nucleotides, which are essential for phage genome replication and completion of its infection cycle (Han et al., 2022). In our study, the differences observed between the water and the feed group may be due to the timing of the infective cycle of the phage due to phage arrival time and bacterial stress (Han et al., 2022). In this sense, phage encapsulation could change the phage pharmacokinetics, not only by protection from chemical stress and prolonged release of phage, but also making phage less visible to the immune system (Dąbrowska, 2019).

The relationship of gut microbiota distortion with the individual’s metabolic state was reported by previous authors, who showed how alterations of the gut microbiota in mice by phage administration affected the host gut metabolic phenotype (Hsu et al., 2019). For example, the microbiota is responsible for transforming complex carbohydrates from the feed into products such as lactate, pyruvate or succinate and short-chain fatty acids; or degrading proteins, leading to the production of amino acids, branched-chain fatty acids, amines and harmful phenolic compounds, among others (Aldars-García et al., 2021). Moreover, the gastrointestinal microbiota could modify host-derived metabolites (such as bile acids or cholesterol) or synthesise *de novo* metabolites (Aldars-García et al., 2021). In this sense, certain bacteria of the phyla *Bacteroidetes* and *Firmicutes* have been related to using amino acid to form short-chain fatty acids (Kumar et al., 2019). The amino acids present in food or those synthesized by the host can be processed for the gut microbiota for protein synthesis (Wu L. et al., 2021b). Indeed, *Streptococcus* is one of the bacteria related to the proteolytic processes (Wu L. et al., 2021b). Thus, the changes observed in genera from these phyla could be related to these altered metabolites. Considering that gut metabolites could not only impact the balance of intestinal microecology but could also regulate anatomically distant biological systems from the gut *via* the bloodstream (Lu et al., 2021; Tomasova et al., 2021), it will be important to shed light and better investigate on all the changes that are taking place.

Our study shows that preventive therapy with bacteriophages minimally alters the intestinal microbiota but significantly impacts their metabolites, regardless of the route of administration. Further studies are needed to understand the potential interplay between differentially abundant bacterial species and significantly altered metabolites to clarify phage treatment implications.

## Data Availability

The data of the microbiota presented in the study are deposited in the NCBI’s repository, accession number BioProject PRJNA876127. The data of the metabolome presented in the study are deposited in the NMDR repository, accession number study ID ST002310.
