# Supplementary material for: Examining the effects of Salmonella phage on the caecal microbiota and metabolome features in Salmonella-free broilers
Source: Front Genet. 2022 Nov 10;13:1060713. doi: 10.3389/fgene.2022.1060713 (PMC9691336; doi:10.3389/fgene.2022.1060713)
Supplement: Supplementary file 1 [file Table1.DOCX]

**Supplementary Table 1:** DADA2 statistics for each group (control, water and feed). Input represents the number of reads used after the trimming and merging/concatenating steps; Filtered is the amount of sequences remaining after DADA2 filtering based on a maxee=2; Denoised is the number of sequences remaining after DADA2 denoising; Merged and % of input merged applies only to pipelines with merging by DADA2 (NMd, LMd , QMd and QdMd); Non-chimeric output sequences were passed into the taxonomic classifier; % output is the percent of sequences from the input left for taxonomic classification.

| **Group** | **Input** | **Filtered** | **% of input passed filter** | **Denoised** | **Merged** | **% of input merged** | **Non-chimeric** | **% of input non-chimeric** |
| --- | --- | --- | --- | --- | --- | --- | --- | --- |
| **Control** | 159083.00 | 109026.13 | 68.43 | 106351.80 | 102343.87 | 64,12 | 97208.07 | 60,94 |
| **Water** | 165713.27 | 112936.67 | 68.15 | 109892.73 | 105280.53 | 63,51 | 99924.53 | 60,36 |
| **Feed** | 144844.47 | 94659.40 | 63.92 | 91908.80 | 87433.60 | 58,78 | 82338.20 | 55,40 |
