# Supplementary material for: Examining the effects of Salmonella phage on the caecal microbiota and metabolome features in Salmonella-free broilers
Source: Front Genet. 2022 Nov 10;13:1060713. doi: 10.3389/fgene.2022.1060713 (PMC9691336; doi:10.3389/fgene.2022.1060713)
Supplement: Supplementary file 2 [file Table2.docx]

**Supplementary Table 2**. Caecal microbiota features characterised for *Salmonella*-free broilers treated with a *Salmonella* phage. Bayesian statistical analysis of the relevant genera identified by partial least square-discriminant analysis (PLS-DA) in water- and feed-phage treated chickens compared with the control group, computed as control *vs* water and control *vs* feed. The water group received a 10^8^ PFU/mL phage concentration via drinking water. The feed group received a 10^8^ PFU/g phage concentration via feed (encapsulated). The control group did not receive a phage.

| **Experimental groups** | **Family** | **Genera** | **HPD95** | **P0** | **D** |
| --- | --- | --- | --- | --- | --- |
| **Control *vs* Water** | *Streptococcaceae* | *Streptococcus* | [-1.34,0.14] | 94.97 | -0.62 |
|  | *Lachnospiraceae* | *Sellimonas* | [-1.34,0.12] | 94.73 | -0.60 |
| **Control *vs* Feed** | *Ruminococcaceae* | *Faecalibacterium* | [-0.52,1.01] | 72.41 | 0.23 |
|  |  | *Ruminococcus* | [-0.55,0.99] | 71.52 | 0.22 |
|  |  | *Incertae_Sedis* | [-0.95,0.58] | 67.74 | -017 |
|  |  | *Ruminococcus_torques_group* | [-1,0.51] | 74.63 | -0.25 |
|  |  | *Ruminococcus_gauvreauii_group* | [-1.04,0.48] | 75.57 | -0.26 |
|  | *Lactobacillaceae* | *Lactobacillus* | [-1.32,0.18] | 93.39 | -0.57 |
|  | *Butyricicoccaceae* | *Butyricicoccus* | [-0.67,0.88] | 58.95 | 0.09 |
|  | *Acholeplasmataceae* | *Anaeroplasma* | [-1.62,-0.17] | 99.11 | -0.89 |
|  | *Clostridia_vadinBB60_group* | *Clostridia_vadinBB60_group* | [-1.45,0.03] | 96.87 | -0.70 |

HPD95%= The highest posterior density region at 95% of probability. P0= Probability of the difference (Dcontrol-water or Dcontrol-feed) being greater than 0 when Dcontrol-water or Dcontrol-feed > 0 or lower than 0 when Dcontrol-water or Dcontrol-feed < 0. D = Mean of the difference control vs water or control vs feed (median of the marginal posterior distribution of the difference between the control group and the water group or feed group). Statistical differences were assumed if | Dcontrol-water | or | Dcontrol-feed | surpass R value and its P0>0.90.
