# Supplementary material for: Examining the effects of Salmonella phage on the caecal microbiota and metabolome features in Salmonella-free broilers
Source: Front Genet. 2022 Nov 10;13:1060713. doi: 10.3389/fgene.2022.1060713 (PMC9691336; doi:10.3389/fgene.2022.1060713)
Supplement: Supplementary file 3 [file Table3.docx]

**Supplementary Table 3**. Caecal microbiota features characterised for *Salmonella*-free broilers treated with a *Salmonella* phage. Bayesian statistical analysis of the relevant metabolite identified by partial least square-discriminant analysis (PLS-DA) in water- and feed-phage treated chickens compared with the control group, computed as control vs water and control vs feed. The water group received a 10^8^ PFU/mL phage concentration via drinking water. The feed group received a 10^8^ PFU/g phage concentration via feed (encapsulated). The control group did not receive a phage.

| **Experimental groups** | **Super class** | **Class** | | **Subclass** | | **Name** | | | **Formula** | **ION** | **HPD95** | **P0** | **D** |
| --- | --- | --- | --- | --- | --- | --- | --- | --- | --- | --- | --- | --- | --- |
| **Control**  **vs**  **Water** | Organic oxygen compounds | Organooxygen compounds | | Carbohydrates and carbohydrate conjugates | | 7-Hydroxy-2-Methyl-4-oxo-4H-1-benzopyran-5-carboxylic acid 7-glucoside | | | C17H18O10 | [M+F]- | [-1.22,0.48] | 81.61 | -0.38 |
|  |  |  |  |  |  | Dimboa glucoside | | | C15H19NO10 | [M+F]- | [-1.57,0.02] | 96.95 | -0.76 |
|  |  |  |  |  |  | D-Glucono-1,5-lactone 6-phosphate | | | C15H14O4 | [M-H]- | [-0.89,0.83] | 55.43 | -0.06 |
|  |  |  |  |  |  | Ribose-1-arsenate | | | C5H11AsO8 | [M-H]- | [-1.15,0.55] | 76.07 | -0.30 |
|  |  |  |  |  | | Chavicol O-beta-glucopyranoside | | | C15H20O6 | [M-H]- | [-0.95,0.72] | 60.60 | -0.11 |
|  |  |  |  | Carbonyl compounds | | Stearyl monoglyceridyl citrate | | | C28H54O12 | [M+Na-2H]- | [-0.12,1.52] | 95.24 | 0.70 |
|  |  |  |  |  |  | 2-Hydroxy-4-methoxyacetophenone 5-sulfate | | | C9H10O7S | [M-H]- | [-0.16,1.47] | 93.64 | 0.63 |
|  | Organic acids and derivates | Hydroxy acids and derivatives | | Beta hydroxy acids and derivatives | | Diethyl L-malate | | | C8H14O5 | [M+H]+ | [-0.45,1.24] | 82.13 | 0.39 |
|  |  | Peptidomimetics | | Hybrid peptides | | L-beta-aspartyl-L-alanine | | | C7H12N2O5 | [M+2Na-H]+ | [-0.06,1.59] | 96.22 | 0.73 |
|  |  | Carboxylic acids and derivates | | Amino acids, peptides and analogues | | Cysteinyl-Hydroxyproline | | | C8H14N2O4S | [M+K]+ | [-0.8,0.91] | 55.54 | 0.07 |
|  |  |  |  |  |  | Tolmentin glucuronide | | | C21H23NO9 | [M+H-2H2O]+ | [-0.08,1.55] | 96.13 | 0.73 |
|  |  |  |  |  |  | Hydroxyphenylacetylglycine | | | C10H11NO4 | [M-H2O-H]- | [-0.2,1.45] | 93.54 | 0.63 |
|  |  |  |  |  |  | Nicotinamide Adenine Dinucleotide Phosphate | | | C21H28N7O17P3 | [M+2H+Na]3+ | [-1.36,0.31] | 90.70 | -0.55 |
|  |  |  |  |  |  | Pentosidine | | | C17H26N6O4 | [M+CH3COO]- | [-0.52,1.16] | 77.44 | 0.32 |
|  |  |  |  |  |  | DL-o-Tyrosine | | | C9H11NO3 | [M+H]+ | [-0.63,1.08] | 70.11 | 0.23 |
|  |  |  |  |  |  | DL-Methionine sulfoxide | | | C5H11NO3S | [M+FA-H]- | [-0.53,1.14] | 74.96 | 0.28 |
|  |  |  |  |  |  | Tauropine | | | C5H11NO5S | [M-H]- | [-0.44,1.24] | 84.61 | 0.42 |
|  |  |  |  |  |  | D-Glutamate | | | C5H9NO4 | [M+H]+ | [-0.99,0.71] | 63.47 | -0.15 |
|  |  |  |  |  | | 2-Aminoheptanoate | | | C7H15NO2 | [M+ACN+H]+ | [-0.38,1.3] | 85.70 | 0.45 |
|  |  |  |  | Carboxylic acid derivates | | N-Acetylcadaverine | | | C7H16N2O | [M+H]+ | [-0.04,1.55] | 97.30 | 0.78 |
|  |  |  |  |  |  | N-(3,4-Dichlorophenyl)-malonamate | | | C9H7Cl2NO3 | [M+NH4]+ | [-1.16,0.54] | 77.44 | -0.32 |
|  |  |  |  | Oxo carboxylic acid | | 4-Oxododecanedioic acid | | | C12H20O5 | [M-H]- | [-1.37,0.33] | 88.97 | -0.52 |
|  | Lipids and lipid-like molecules | Prenol lipids | | Quinone and hydroquinone lipid | | 7C-aglycone | | | C18H18O4 | [M-H]- | [-1.36,0.3] | 89.39 | -0.51 |
|  |  |  |  | Monoterpenoids | | Monomenthyl succinate | | | C14H24O4 | [M+FA-H]- | [-0.2,1.47] | 93.64 | 0.64 |
|  |  | Fatty Acyls | | Eicosanoid | | PGA3 | | | C20H28O4 | [M-H]- | [-1.55,0.1] | 95.40 | -0.70 |
|  |  |  | | Fatty acids and conjugates | | 2-Hydroxyoctadecanoic acid | | | C18H36O3 | [M-H]- | [-0.78,0.94] | 58.20 | 0.09 |
|  | Lignans, neolignans and related compounds | Furanoid lignans | | Tetrahydrofuran lignans | | Enterolactone | | | C18H18O4 | [M+FA-H]- | [-1.2,0.48] | 81.33 | -0.37 |
|  | Benzenoids | Phenols | | Methoxyphenols | | Vanylglycol | | | C18H36O4Si3 | [M-H]- | [-0.77,0.94] | 57.77 | 0.09 |
|  |  |  | | Phenol ethers | | Dictagymnin | | | C14H18O | [M+H-H2O]+ | [-0.13,1.53] | 95.85 | 0.73 |
|  |  | Benzene and substituited derivates | | Benzene and substituited derivates | | 4-Methyl-1-phenyl-2-pentanone | | | C12H16O | [M+H-H2O]+ | [-0.12,1.55] | 95.71 | 0.72 |
|  | Phenylpropanoids and polyketides | Isoflavonoids | | Isoflavans | | 3'-Hydroxyequol | | | C15H14O4 | [M-H]- | [-0.5,1.2] | 79.78 | 0.36 |
|  |  |  | | O-methylated isoflavonoids | | Isosativan | | | C17H18O4 | [M-H]- | [-1.17,0.5] | 78.46 | -0.33 |
|  |  | Cinnamic acids and derivatives | | Hydroxycinnamic acids and derivatives | | (R)-2-Feruloyl-1-(4-Hydroxyphenyl)-1,2-ethanediol | | | C18H18O6 | [M-H]- | [-1.68,-0.15] | 98.93 | -0.92 |
|  |  |  |  |  |  | Avenanthramide A2 | | | C18H19NO7 | [M-H]- | [-1.18,0.49] | 80.39 | -0.35 |
|  |  | Phenylpropanoids and polyketides | | Phenylpropanoids acids | | 3-(3,5-Diiodo-4-hydroxyphenyl)lactate | | | C9H8I2O4 | [M+FA-H]- | [-1.2,0.51] | 80.03 | -0.35 |
|  |  | Kavalactones | | Kavalactones | | 5,6-Dihydro-11-methoxyyangonin | | | C16H18O5 | [M-H2O-H]- | [-1.42,0.26] | 90.94 | -0.56 |
|  | Non-identified metabolite 150 | | | | | | | | | | [-1.48,0.19] | 93.99 | -0.66 |
|  | Non-identified metabolite 152 | | | | | | | | | | [-1.44,0.22] | 93.34 | -0.63 |
|  | Non-identified metabolite 203 | | | | | | | | | | [-1.68,-0.09] | 98.67 | -0.89 |
|  | Non-identified metabolite 213 | | | | | | | | | | [-1.57,0.02] | 97.29 | -0.77 |
|  | Non-identified metabolite 232 | | | | | | | | | | [-0.81,0.94] | 58.55 | 0.09 |
|  | Non-identified metabolite 233 | | | | | | | | | | [-1.57,0] | 97.59 | -0.79 |
|  | Non-identified metabolite 244 | | | | | | | | | | [-0.59,1.15] | 75.66 | 0.30 |
|  | Non-identified metabolite 273 | | | | | | | | | | [-1.94,-0.57] | 99.96 | -1.25 |
|  | Non-identified metabolite 277 | | | | | | | | | | [-1.04,0.64] | 67.04 | -0.19 |
|  | Non-identified metabolite 326 | | | | | | | | | | [-0.03,1.62] | 97.07 | 0.79 |
|  | Non-identified metabolite 400 | | | | | | | | | | [0.21,1.78] | 99.35 | 1.00 |
|  | Non-identified metabolite 514 | | | | | | | | | | [-0.55,1.16] | 77.81 | 0.33 |
|  | Non-identified metabolite 566 | | | | | | | | | | [-0.3,1.37] | 91.27 | 0.57 |
|  | Non-identified metabolite 568 | | | | | | | | | | [-0.08,1.56] | 95.68 | 0.71 |
|  | Non-identified metabolite 571 | | | | | | | | | | [-0.35,1.33] | 88.98 | 0.51 |
|  | Non-identified metabolite 572 | | | | | | | | | | [-0.09,1.56] | 96.34 | 0.76 |
|  | Non-identified metabolite 573 | | | | | | | | | | [-0.3,1.35] | 90.15 | 0.54 |
|  | Non-identified metabolite 595 | | | | | | | | | | [-0.74,0.96] | 58.66 | 0.10 |
|  | Non-identified metabolite 637 | | | | | | | | | | [-1.31,0.38] | 85.93 | -0.46 |
|  | Non-identified metabolite 644 | | | | | | | | | | [-1.23,0.46] | 82.57 | -0.39 |
|  | Non-identified metabolite 647 | | | | | | | | | | [-1.3,0.34] | 87.08 | -0.47 |
|  | Non-identified metabolite 648 | | | | | | | | | | [-1.28,0.39] | 86.12 | -0.46 |
|  | Non-identified metabolite 669 | | | | | | | | | | [-0.86,0.88] | 51.02 | 0.02 |
|  | Non-identified metabolite 678 | | | | | | | | | | [-0.04,1.6] | 97.16 | 0.80 |
|  | Non-identified metabolite 683 | | | | | | | | | | [-0.56,1.16] | 75.82 | 0.30 |
|  | Non-identified metabolite 698 | | | | | | | | | | [-0.62,1.08] | 70.59 | 0.23 |
|  | Non-identified metabolite 705 | | | | | | | | | | [-1.03,0.67] | 69.12 | -0.21 |
|  | Non-identified metabolite 712 | | | | | | | | | | [-1.19,0.49] | 79.47 | -0.34 |
|  |  | | | | | | | | | |  |  |  |
| **Control**  **vs**  **Feed** | Organic oxygen compounds | | Organooxygen compounds | | Carbohydrates and carbohydrate conjugates | | Dimboa glucoside | C15H19NO10 | | [M+F]- | [-1.47,0.1] | 96.08 | -0.70 |
|  |  |  |  |  | Carbonyl compounds | | 4-(2-Aminophenyl)-2,4-dioxobutanoic acid | C10H9NO4 | | [M+H]+ | [-1.44,0.16] | 93.74 | -0.63 |
|  | Organic acids and derivates | | Carboxylic acids and derivates | | Amino acids, peptides and analogues | | L-Agaritine | C12H17N3O4 | | [M+K-2H]- | [0.17,1.71] | 99.23 | 0.96 |
|  | Lipids and lipid-like molecules | | Steroids and steroid derivates | | Sulfated steroids | | Androsterone sulfate | C19H30O5S | | [M+CH3COO]- | [-1.54,0.04] | 96.57 | -0.74 |
|  |  |  | Prenol lipids | | Quinone and hydroquinone lipid | | 7C-aglycone | C18H18O4 | | [M-H]- | [-1.48,0.12] | 95.20 | -0.68 |
|  | Phenylpropanoids and polykeides | | Cinnamic acids and derivatives | | Hydroxycinnamic acids and derivatives | | (R)-2-Feruloyl-1-(4-Hydroxyphenyl)-1,2-ethanediol | C18H18O6 | | [M-H]- | [-1.65,-0.12] | 98.86 | -0.90 |
|  | Non-identified metabolite 154 | | | | | | | | | | [0.59,1.9] | 99.97 | 1.24 |
|  | Non-identified metabolite 203 | | | | | | | | | | [-1.5,0.05] | 96.43 | -0.71 |
|  | Non-identified metabolite 213 | | | | | | | | | | [-1.61,-0.06] | 98.63 | -0.88 |
|  | Non-identified metabolite 233 | | | | | | | | | | [-1.64,-0.11] | 98.85 | -0.89 |
|  | Non-identified metabolite 273 | | | | | | | | | | [-2.04,-0.7] | 99.98 | -1.36 |
|  | Non-identified metabolite 400 | | | | | | | | | | [-0.12,1.41] | 94.89 | 0.63 |
|  | Non-identified metabolite 420 | | | | | | | | | | [-0.04,1.54] | 96.65 | 0.75 |
|  | Non-identified metabolite 557 | | | | | | | | | | [-0.16,1.46] | 94.79 | 0.66 |

HPD95%= The highest posterior density region at 95% of probability. P0= Probability of the difference (Dcontrol-water or Dcontrol-feed) being greater than 0 when Dcontrol-water or Dcontrol-feed > 0 or lower than 0 when Dcontrol-water or Dcontrol-feed < 0. D = Mean of the difference control vs water or control vs feed (median of the marginal posterior distribution of the difference between the control group and the water group or feed group). Statistical differences were assumed if | Dcontrol-water | or | Dcontrol-feed | surpass R value and its P0>0.90.
